# Knowledge and Awareness of Leukemia Among the Population of Eastern Province, Saudi Arabia

**DOI:** 10.7759/cureus.46382

**Published:** 2023-10-02

**Authors:** Naushad Abid, Abdullah H Bohamad, Hussain I Aljohar, Batla S Al Battat, Yousef Y Altaher, Abdulaziz E Alateeq, Maryam O Alarfaj, Meataz Aljeezan, Ali S AlBashrawi, Ahmed Al Jizan‎

**Affiliations:** 1 Internal Medicine, College of Medicine, King Faisal University, Al-Ahsa, SAU; 2 Internal Medicine and Rheumatology, King Faisal University, Al-Ahsa, SAU; 3 Medicine, King Faisal University, Al-Ahsa, SAU

**Keywords:** awareness, blood cancer, blood pathology, malignancy, leukemia, cancer

## Abstract

Introduction: Cancer is characterized by the rapid proliferation of abnormal cells that exceed their normal boundaries, infiltrating other body parts and leading to metastasis, a distinctive feature setting it apart from other diseases. Metastasis is the primary cause of cancer-related deaths, with nearly 10 million global fatalities, making it the leading cause of mortality. Leukemia, a type of cancer originating in the bone marrow or blood cells, presents significant complications and is associated with various risk factors, including a positive family history, smoking, and obesity. This study aims to evaluate the general knowledge of leukemia and its associated risks among the inhabitants of the Eastern Province, Saudi Arabia.

Methods: A cross-sectional survey was conducted targeting* *all residents of the Eastern Province, Saudi Arabia, who were Saudi nationals, spanning both genders and aged 15 to 59 years. The questionnaire was distributed electronically through social networking applications, and responses were collected via Google Forms (Google, Mountain View, CA).

Results: The study findings indicated that the most frequently identified risk factors for leukemia included blood disorders, genetic disorders, and extensive exposure to radiation and chemicals. The most prevalent leukemia symptoms were bruising and bleeding, followed by vomiting, nausea, and headache. The most commonly reported side effects of leukemia treatment were dizziness, followed by anorexia and exhaustion.

Conclusion: The study revealed a lack of awareness about leukemia and its associated risks among participants. This underscores the need for continued educational initiatives and awareness campaigns to improve leukemia knowledge and early detection rates, potentially leading to better outcomes for affected individuals. Future research should aim to overcome study limitations and provide a broader perspective on leukemia awareness throughout Saudi Arabia.

## Introduction

The rapid development of aberrant cells that outgrow their normal bounds, subsequently infecting other body parts and spreading to other organs, is one of the characteristics that distinguishes cancer from other diseases [1,2]. Almost 10 million people died from cancer worldwide in 2020, making it the leading cause of death [1]. In terms of new cancer cases in 2020, the most prevalent were breast, lung, colon, and rectal cancers. Moreover, breast, colorectal, prostate, brain, and lymphoma cancers are the most common in Saudi Arabia [3,4]. According to the Saudi Cancer Registry, in 2017, leukemia ranked fifth among all cancers affecting Saudis of all ages and genders. In the Saudi population, the overall prevalence of leukemia was 7.6% for males and 4.4% for females [5,6]. Through cancer prevention and early detection measures, it is possible to reduce the overall cancer burden and increase survival rates [4]. Leukemia is a type of cancer that develops in the bone marrow or blood cells and causes significant complications [7]. Leukemia is associated with various risk factors, such as positive family history, smoking, obesity, and exposure to benzene, radiation, and chemotherapy [8]. Approximately 437,033 new cases of leukemia were diagnosed in 2020, while 309,006 individuals died from the disease [9]. The management of leukemia includes chemotherapy, biological therapy, radiation, and bone marrow transplantation [7]. A study conducted in Saudi Arabia showed that most participants had a low level of knowledge regarding different forms of leukemia. Only 13.4% of participants had a strong understanding of leukemia and its types [10]. This study aims to assess the level of awareness among the general population regarding leukemia in the Eastern region of Saudi Arabia.

## Materials and methods

Study design

This was a descriptive cross-sectional study conducted among the population of the Eastern region of Saudi Arabia. A self-administered questionnaire was distributed using an online social platform to investigate awareness of leukemia.

Inclusion and exclusion criteria

The inclusion criteria were Saudi Arabian citizens from the Eastern region, aged older than 15 years, and willing to participate. The exclusion criteria were non-Saudi Arabian citizens, individuals who did not reside in the Eastern region, those younger than 15 years old, and those not willing to participate.

Data collection methods and tools

This study used online questionnaires that were distributed via social media platforms such as WhatsApp, Snapchat, and Telegram to gather data. A structured questionnaire was developed based on extensive literature. The questionnaire was divided into three sections: informed consent was included in the first section; questions about socioeconomic status, such as age, gender, educational level, and marital status, were included in the second section; questions measuring knowledge about leukemia and its risk factors were included in the last section.

Data analysis

The data collected from the online questionnaires were stored and analyzed using Statistical Package for Social Sciences (SPSS) version 26 (IBM Corp., Armonk, NY).

Research ethics approval and ethical consideration

The research was approved by the Ethics Committee of King Faisal University (code number: KFU-REC-2023-MAY-ETHICS906). Before proceeding to the questionnaire, participants were provided with an assurance that their private information and confidentiality would be safeguarded. Participating in and submitting the questionnaire indicated their consent to be part of the study.

## Results

The results showed that there were 517 participants, with the majority being female (68.5%) and 31.5% male. In terms of education, 63.4% had a bachelor's degree or diploma, 27.9% had a secondary education, 4.4% had an intermediate level of education, 3.3% had a master's degree or PhD, 0.8% had primary education, and 0.2% were illiterate. Regarding their ages, 80.3% were aged between 18 and 30 years, 9.7% were aged between 31 and 40 years, 5.2% were aged between 41 and 50 years, and 4.8% were aged over 50 years. In terms of marital status, 73.5% were single, 24.2% were married, 2.1% were divorced, and 0.2% were widowed (Table 1).

**Table 1:** Demographic characteristics

Variables	Categories	N	%
Gender	Male	163	31.5
	Female	354	68.5
Education	Illiterate	1	0.2
	Primary	4	0.8
	Intermediate	23	4.4
	Secondary	144	27.9
	Bachelors/diploma	328	63.4
	Masters/PhD	17	3.3
Age	From 18 to 30 years	415	80.3
	From 31 to 40 years	50	9.7
	From 41 to 50 years	27	5.2
	More than 50 years	25	4.8
Marital status	Single	380	73.5
	Married	125	24.2
	Divorced	11	2.1
	Widowed	1	0.2

The results showed that the most frequent side effects of leukemia treatment were dizziness (66.5%), followed by anorexia (63.6%), exhaustion (59.6%), poor focus (57.8%), easy bruising (50.5%), oral ulceration (32.7%), thirst (30.9%), memory loss (21.9%), and hyperactivity (15.5%) (Table 2).

**Table 2:** The side effects of leukemia treatment

Side effects	N	%
Anorexia	329	63.6
Dizziness	344	66.5
Poor focus	299	57.8
Thirst	160	30.9
Exhaustion	308	59.6
Oral ulceration	169	32.7
Easy bruising	261	50.5
Memory loss	113	21.9
Hyperactivity	80	15.5

The results showed that the most frequent symptoms of leukemia were bruising and bleeding (61.1%), followed by vomiting and nausea (52.8%), headache (49.9%), swollen lymph nodes (45.1%), blurred vision (43.9%), osteoporosis (43.3%), frequent infections (39.3%), memory loss (19.5%), and obesity (15.7%) (Table 3).

**Table 3:** The symptoms of leukemia

Symptoms	N	%
Frequent infection	203	39.3
Vomiting and nausea	273	52.8
Osteoporosis	224	43.3
Headache	258	49.9
Blurred vision	227	43.9
Bruising and bleeding	316	61.1
Swollen lymph nodes	233	45.1
Obesity	81	15.7
Memory loss	101	19.5

The results showed that the most frequent risk factors for leukemia were blood disorders (68.7%), genetic disorders (65%), extensive exposure to radiation and chemicals (64.8%), smoking (44.7%), contact with an infected person and fever (18%), consuming fast food (16.1%), contact with an infected person (14.5%), and seasonal diseases (13.9%) (Table 4).

**Table 4:** The risk factors for leukemia

Risk factors	N	%
Blood disorder	355	68.7
Genetic disorder	336	65
Extensive exposure to radiation and chemicals	335	64.8
Fever	93	18
Smoking	231	44.7
Contact with an infected person and fever	93	18
Contact with an infected person	75	14.5
Seasonal diseases	72	13.9
Eat fast food	83	16.1

The results showed a significant association between side effects and gender (χ² = 43.973, P-value < 0.001), education (χ² = 117.303, P-value < 0.001), age (χ² = 48.287, P-value = 0.007), and marital status (χ² = 56.565, P-value < 0.001). There was also a significant association between symptoms and gender (χ² = 61.018, P-value < 0.001), education (χ² = 78.58, P-value = 0.001), and age (χ² = 451.685, P-value = 0.003). Additionally, an association was found between side effects and gender (χ² = 21.135, P-value = 0.012), education (χ² = 120.823, P-value < 0.001), and marital status (χ² = 40.9, P-value = 0.042) (Table 5).

**Table 5:** The factors associated with awareness about cancer (side effects, symptoms, and risk factors)

		Gender	Education	Age	Marital status
Side effects	Chi-square	43.973	117.303	48.287	56.565
	P-value	<0.001	<0.001	0.007	<0.001
Symptoms	Chi-square	61.018	78.58	51.685	36.084
	P-value	<0.001	0.001	0.003	0.113
Risk factors	Chi-square	21.135	120.823	30.221	40.9
	P-value	0.012	<0.001	0.304	0.042

## Discussion

Leukemia is one of the top five cancer types identified in Saudi Arabia. It is associated with blood-forming tissues, including bone marrow and the lymphatic system. In this study, we assessed the general knowledge of the Eastern region's population about leukemia. Regarding the risk factors for leukemia, the results showed that almost two-thirds of participants believed that the most common risk factors were anorexia and dizziness, while memory loss and hyperactivity were selected least frequently. Regarding leukemia symptoms, almost two-thirds of participants believed that bruising and bleeding were the most common symptoms, and half of them believed that vomiting, nausea, headache, and swollen lymph nodes were the most common symptoms. The most common risk factors for leukemia were blood disorders, followed by genetic disorders and extensive exposure to radiation and chemicals.

The results revealed that participants chose dizziness and anorexia as the most frequent side effects of leukemia treatment, while memory loss and hyperactivity were chosen the least. This is very similar to another study where participants selected anorexia as the most frequent side effect of leukemia treatment, and hyperactivity was the least chosen [11]. This suggests that the general population is aware of the side effects of chemotherapy and leukemia management.

Regarding leukemia symptoms, participants believed that bruising and bleeding were the most frequent symptoms, followed by vomiting, nausea, headache, and swollen lymph nodes, while obesity was the least chosen symptom. According to Katherine Tarlock's review, bleeding and bruising were significant symptoms of leukemia, indicating good knowledge. However, nausea and vomiting are not symptoms of leukemia [12].

Regarding risk factors for leukemia, the results showed that the majority of participants believed that the most frequent risk factors for leukemia were blood disorders, followed by genetic disorders, extensive exposure to radiation, and chemicals. We found that the general population was aware of the risk factors for leukemia, which goes against our initial hypothesis. When compared with other research conducted in the north of Saudi Arabia, they found little awareness of leukemia among the population [13]. However, there was low awareness among the public in other countries such as the United Kingdom [14]. Despite the increasing prevalence of leukemia in Saudi Arabia, more research studies and campaigns are needed to increase awareness of leukemia [15]. There was a relatively good awareness level of leukemia in the United States due to continuous campaigns and increasing research studies [16].

Limitations

The study's participants were primarily from the Eastern region of Saudi Arabia, which may not represent the broader Saudi population. This regional focus could introduce sampling bias and limit the generalizability of the findings to the entire country. The study targeted participants aged 15 to 59 years, which excludes younger individuals and older adults. Leukemia can affect individuals of all ages, and the exclusion of these age groups might overlook valuable insights into awareness and knowledge among those populations. The distribution of the questionnaire through social media platforms may have led to a biased sample, as it relies on individuals who are active on these platforms. This could exclude segments of the population with limited internet access or digital literacy. The study relies on self-reported data from participants, which may introduce response bias and inaccuracies. Participants might provide socially desirable responses or misinterpret certain questions. While the study collects some socioeconomic data, it does not delve deeply into participants' socioeconomic backgrounds, which could be important for understanding how awareness of leukemia varies among different social and economic groups.

Recommendations

Future research should aim for a more diverse sample that includes participants from different regions of Saudi Arabia and a wider age range. This would provide a more comprehensive understanding of leukemia awareness across the country. To address potential bias associated with online surveys, researchers should consider conducting face-to-face surveys or phone interviews to include individuals who may not have internet access or use social media. Future research should explore the influence of socioeconomic factors in greater depth to understand how income, education, and other variables impact awareness and knowledge of leukemia.

## Conclusions

This study aimed to assess the general knowledge and awareness of leukemia and its associated risk factors among the population of the Eastern Province, Saudi Arabia. The study involved a diverse group of participants, and the findings showed a suboptimal level of awareness regarding leukemia and its associated risk factors in the region. This highlights the importance of ongoing educational programs and awareness campaigns to enhance knowledge about leukemia and improve early detection rates. Increasing awareness can contribute to better outcomes for individuals affected by leukemia. Future research should aim to address the limitations of this study and provide a more comprehensive understanding of leukemia awareness across Saudi Arabia.
